# Integrative analysis of co-expression networks and codon usage bias in maize under biotic stress

**DOI:** 10.1371/journal.pone.0317755

**Published:** 2025-07-23

**Authors:** Zahra Zinati, Leyla Nazari

**Affiliations:** 1 Department of Agroecology, College of Agriculture and Natural Resources of Darab, Shiraz University, Shiraz, Iran; 2 Crop and Horticultural Science Research Department, Fars Agricultural and Natural Resources Research and Education Center, Agricultural Research, Education and Extension Organization (AREEO), Shiraz, Iran.; Jashore University of Science and Technology, BANGLADESH

## Abstract

Understanding the complex networks underlying the biotic stress response in maize is crucial for developing effective approaches to improve tolerance. We identified 1449 differentially expressed genes (DEGs) by meta-analysis of the public microarray gene expression profile. Weighted Gene Co-expression Network Analysis on the DEGs resulted in positive module-trait correlation (0.71, 0.69, 0.58, and 0.46) in the brown, grey, blue, and green modules, respectively, and negative correlation in the turquoise module. The module membership (MM) and gene significance (GS) were strongly correlated (0.65 and 0.6) in the brown and grey modules, respectively. The enrichment in diterpene phytoalexin and diterpenoid biosynthetic process suggests the involvement of the brown module in the synthesis of compounds necessary for the defense against pathogens. For the grey module, the significant GO terms were related to lipid oxidation, oxylipin, and fatty acid biosynthetic process. Identification of DEGs encoding transcription factors revealed that the MYB, NAC, WRKY, and C2C2 families had the highest membership, each with six members. Noteworthy genes identified include zealexin A1 synthase, CPP synthase, linoleate 9S-lipoxygenase3 (lox3), linoleate 9S-lipoxygenase1 (lox1), and MYB8, were among the top 5% genes with the highest GS and MM values in the brown and grey modules. Codon usage analysis revealed specific preferences under biotic stress, characterized by high Codon Adaptation Index (CAI) and Relative Synonymous Codon Usage (RSCU) values, suggesting an adaptive mechanism for efficient translation and gene regulation during stress. This comprehensive study identified potential targets for genetic engineering aimed at optimizing gene expression for improved stress tolerance.

## Introduction

Maize is a fundamental crop in worldwide farming, serving a dual role as both a key subject for genetic studies and an essential food source. However, its yield is at significant risk due to biotic challenges such as illnesses and pest infestations that can substantially reduce harvests. Managing biotic stresses in maize, such as pests and diseases, can lead to considerable economic expenses as they negatively impact the crop’s productivity. The need to breed maize for resistance to these biotic stresses and study the genetics of resistance reflects the importance of managing these issues to minimize economic losses [1]. The International Maize and Wheat Improvement Center (CIMMYT) is actively involved in creating superior tropical maize germplasm that can withstand crucial abiotic and biotic stress factors. This effort has resulted in the successful release of maize varieties tolerant to these stresses in different areas [2].

Transcription factors are at the heart of plant defense signaling pathways. They regulate the expression of genes involved in the plant’s response to biotic stress, such as pathogen invasion. This regulation is crucial for activating the plant’s immune response, which enables it to counteract stress and minimize damage effectively. For instance, WRKY transcription factors are known to play a significant role in plant defense against pathogens [3–6]. Identifying these TFs in maize allows for a deeper understanding of the molecular mechanisms underlying plant immunity. It offers potential targets for genetic engineering or breeding programs to enhance disease resistance. For instance, the overexpression of ZmWRKY65, a WRKY transcription factor in maize, has been demonstrated to confer increased resistance to both pathogen attack and drought stress in transgenic Arabidopsis plants [4].

Although previous research sheds light on genetic mechanisms associated with stress tolerance in maize, there are gaps that need to be addressed. Previous studies often focused on individual genes or pathways, overlooking the complex network of gene interactions that underlie stress responses. The advancement of next-generation sequencing technology offers a unique opportunity to analyze complex biological systems in depth [7]. WGCNA (Weighted Gene Co-expression Network Analysis) is an effective method in systems biology that allows researchers to investigate the intricate relationships among genes and how they relate to biological functions and traits [8,9]. The aim of this study is to fill this gap by using Weighted Gene Co-expression Network Analysis (WGCNA) to elucidate the co-expression patterns of genes involved in the defense of maize against biotic stress. WGCNA has been applied to discover groups of genes that are both functionally associated and co-expressed, which change in regulation due to various biotic stresses in Arabidopsis. This method is used to build an undirected network made up of distinct clusters of co-expressed genes, which helps uncover the genetic foundation of stress regulation and pinpoint crucial hub genes that could act as central regulators in the plant’s response to diseases [10]. WGCNA has also been employed to identify sets of genes with similar expression profiles that are highly interrelated within various metabolic networks, offering insights into the regulatory networks in maize as it responds to abiotic stresses [11]. WGCNA has been utilized to categorize differentially expressed genes into modules that include hub genes, clarifying the molecular mechanisms specific to different growth stages that control maize’s response to drought stress. By analyzing network topology, it is possible to identify key genes within and between modules, which can then be targeted in reverse genetic experiments to dissect further the plant’s immune system [12]. In our previous research, a meta-analysis was conducted on the maize gene expression profiles under different biotic stresses caused by pests or fungal pathogens. This analysis aimed to pinpoint crucial genes associated with tolerance, ultimately unveiling the genetic determinants responsible for maize tolerance to biotic stress through the application of Correlation-based Feature Selection (CFS) [13].

While feature selection improved our understanding of Differentially Expressed Genes (DEGs), the complexity of genetic interplay and regulation remains only partially understood. The use of WGCNA allows us to move beyond individual genes to understand the collective response of gene networks, providing a more holistic view of maize defense strategies.

Through this research, we aim to elucidate the complex gene networks underlying the biotic stress response in maize. We compiled microarray gene expression datasets from 10 relevant studies, comprising a total of 142 samples. By performing a meta-analysis on this comprehensive dataset, we identified DEGs associated with biotic stress in maize. We then used the power of WGCNA to uncover the co-expression modules within the DEG dataset. This approach allowed us to determine the module membership (MM) and gene significance (GS) of each gene in relation to the biotic stress response. To further characterize the key co-expression modules, we conducted a functional enrichment analysis using the bioinformatics tool DAVID. This analysis revealed the biological processes and signaling pathways that were significantly associated with the top 5% of genes with the highest values in both GS and MM in the modules identified by WGCNA. In addition, transcription factors involved in biotic stress responses in maize were identified. By integrating these multi-faceted analyses, the study provides a detailed overview of the complex gene networks and underlying mechanisms that govern the biotic stress response in maize.

Codon usage bias can influence gene expression levels, protein folding, and overall cellular efficiency, thereby impacting the plant’s ability to respond to stress. By analyzing the codon usage of DEGs identified in our study, we aimed to determine whether specific codons are preferentially used in stress-responsive genes. This analysis can reveal adaptive strategies employed by maize at the translational level to optimize protein synthesis under stress conditions. Understanding and leveraging codon usage patterns can aid in the development of genetically engineered plants with improved stress tolerance, contributing to agricultural resilience and productivity. Additionally, to better understand how codon usage bias might influence gene expression and contribute to the maize response to stress, we explored the relationship between codon usage patterns and the significance of gene co-expression modules involved in maize response to biotic stress.

## Materials and methods

### Data collection, preprocessing, and DEG finding

Gene expression profiles related to biotic stress in maize in CEL format based on Affymetrix platform GPL4302 were retrieved from the NCBI Gene Expression Omnibus database (GEO, https://www.ncbi.nlm.nih.gov/geo/). The following datasets including GSE48536, GSE31188, GSE48406, GSE29747, GSE40052, GSE27626, GSE19559, GSE19501, GSE10023, and GSE12892 were fetched. Employing the GEOquery package in R version 4.1.2., raw expression datasets were downloaded and were quantile normalized using Robust Multichip Average (RMA) in the affy Bioconductor package [14]. Afterward, we merged the datasets and removed the batch effect among datasets using the ComBat function of the SVA R package (version 3.54.0) [15]. Then, DEGs were identified using the limma package (version 3.60.6) with the FDR (False Discovery Rate) set to 0.05.

### Weighted gene co-expression network analysis

A WGCNA network was established for the DEGs values employing the WGCNA package (version 1.73) in r to discover the significant modules in response of maize to biotic stress. To achieve this aim, the DEGs matrix of 88 samples including 57 stresses and 31 controls was used to employ weighted gene correlation network analysis (Supplementary file; S1 Table). Briefly, a similarity matrix (Sij) was built using Pearson correlation according to the following formula:

Sij = |0.5 + 0.5*cor (xi, xj)|

Then, it was converted into an adjacency matrix [Aij = (|0.5 + 0.5 *cor (xi, xj)|)β], where β as soft-thresholding power was adjusted to 18. Afterward, we developed a topological overlap similarity measure (TOM) out of the adjacency matrix. Modules were constructed out of TOM using the dynamic tree cut algorithm [16], adjusting a deep split level of 2, a height of 0.15, and a minimum module size of 40.

### Module membership (MM) and gene significance (GS) analysis

Gene significance (GS) represents the gene’s association with the stress condition, while module membership (MM) refers to the gene’s correlation with a specific module. Notably, a high degree of correlation between MM and suggests that the genes within a module are not only functionally related but also coordinately responding to the stress condition, providing valuable insights into the module’s role in mediating the stress response [17]. For each module, GS and MM were calculated and the top 5% of genes exhibiting the highest values in both GS and MM were selected for further analysis in the modules showing a high and significant correlation between GS and MM.

### Gene ontology and pathway annotation of significant modules

To gain a deeper understanding of the functions and processes of the top 5% of genes exhibiting the highest values in both GS and MM within the significant module, gene ontology and pathway annotation were conducted using DAVID (https://david.ncifcrf.gov/) with the given parameters. Using the KEGG database [18], which is integrated into the DAVID bioinformatics resources, we performed the pathway enrichment analyses.

### Identification of DEGs encoding transcription factors

To determine TFs and classify individual TFs into different gene families, we retrieved the protein sequences of the differential genes from the Ensembl Plants (http://plants.ensembl.org), and the sequences were blasted against the database of iTAK with an E-value cutoff of 10−5.

### Validation of key genes using xgboost

Package ‘xgboost’ version 1.6.0.1 was employed to validate genes identified by integration of WGCNA and gene ontology enrichment and transcription factor analysis. XGBoost (eXtreme Gradient Boosting) [19] is a machine learning algorithm based on gradient boosting trees that is found to be an effective method for a variety of tasks including ranking, regression, and classification. A cross-validation was performed using 75% and 25% data in the training and test set and the number of XGBoost rounds was set to 50. The importance of each gene in the model was determined.

### Codon usage analysis

To understand the efficiency of translational and the mechanisms of regulation of gene expression under biotic stress conditions, codon usage patterns of DEGs were analyzed. The analysis was performed using the R programming language with several bioinformatics packages, including coRdon (version 1.22.0) and cubar (version 0.5.0).

The DEG sequences were loaded from a FASTA file and checked for their validity as coding sequences (CDS). Codons within the DEG sequences were counted to facilitate subsequent analyses. Relative synonymous codon usage (RSCU) was calculated to assess codon bias. Codons with RSCU values greater than 1.0 are used more frequently than expected by chance, indicating a bias in their usage [20]. The Codon adaptation index (CAI) was computed to evaluate the adaptability of codon usage of DEGs to the preferred codon usage of highly expressed genes. Higher CAI values indicate better adaptation and potentially higher expression levels [21]. The effective number of codons (ENC) was calculated to measure codon usage bias. Values range from 20 (extreme bias, only one codon used per amino acid) to 61 (no bias, all codons used equally) [21]. GC content, an important indicator of gene composition and stability, was calculated. Higher GC content can indicate higher stability of the DNA molecule [22]. GC content at the third codon position (GC3S), which is indicative of codon usage preference, was calculated. High GC3S values suggest a preference for GC-rich codons at the third position [23]. The calculated indices, including RSCU, CAI, ENC, GC content, and GC3S, were crucial in understanding the codon usage bias and its potential impact on gene expression and stress response.

Moreover, to investigate the relationship between module significance and codon usage patterns, we performed a correlation analysis between codon usage metrics CAI, ENC, GC content, and GC3S and the eigengenes (MEs) of the significant modules, as well as trait-module relationships. This analysis was conducted using R software, with the Hmisc package for Pearson correlation and p-value calculations and the ggplot2 and reshape2 packages for visualization of the correlation matrix.

## Results

### DEGs identification

We conducted a comprehensive meta-analysis of gene expression responses in maize to biotic stress, integrating data from 10 studies encompassing 142 samples. After rigorous pre-processing and batch effect correction, we identified 1449 DEGs between control and biotic stress conditions (adjusted p-value < 0.05) (supplementary file; S1 Table).

### Co-expression gene network and hub gene identification

**Fig 1:**
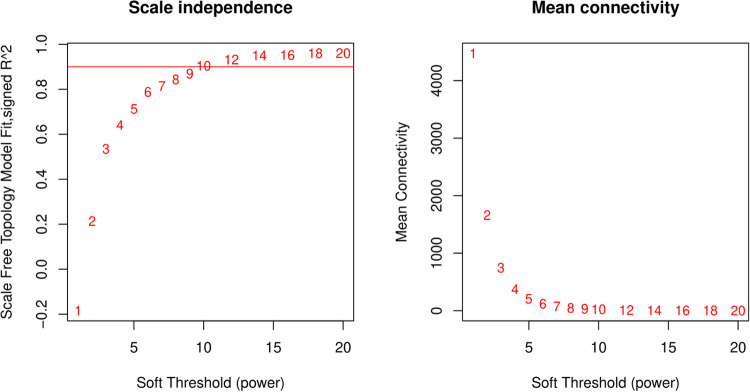
Analysis of network topology for various soft-thresholding powers. These plots are used to determine the soft-thresholding power in a network analysis, which affects the strength of the correlation between genes.

The gene expression profile of 1449 DEGs was used as input to the WGCNA package. In the scale independence plot on the left, the y-axis represents the scale-free fit index (R²), and the x-axis displays the soft-thresholding power. The red line in the plot indicates the threshold for the scale-free topology criterion, typically set to 0.9. This criterion helps to ensure that the network conforms to a scale-free topology. The numbers above the plot points indicate the corresponding soft-thresholding power. The mean connectivity plot on the right shows the average connectivity of the network nodes, which decreases as the soft-thresholding power increases. This plot helps to avoid choosing a power that is too high, which would lead to a disconnected network. In our analysis, the scale-free topology fit appears to reach the desired threshold at a soft-thresholding power of 14 (Fig 1).

**Fig 2:**
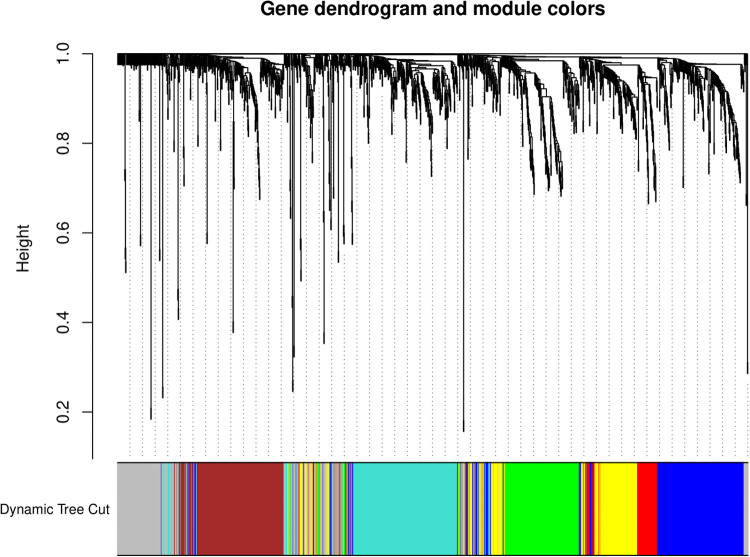
Gene dendrogram and module colors. This dendrogram shows the clustering results obtained using the “average” method on the “dissTOM” distance matrix. The horizontal axis represents the samples, and the vertical axis represents the clustering height. The height on the vertical axis indicates the level of dissimilarity between the modules, calculated based on gene expression similarity.

We then applied hierarchical clustering to analyze the gene expression data, resulting in a comprehensive gene dendrogram. This dendrogram reveals the relationships among genes based on their expression patterns. By employing the Dynamic Tree Cut method, we identified distinct gene modules, each denoted by a unique color (Fig 2). These modules represent clusters of co-expressed genes, indicating a potential functional linkage or shared regulatory mechanisms. The analysis successfully categorized genes into several modules, demonstrating the underlying genetic architecture contributing to the observed traits.

**Fig 3:**
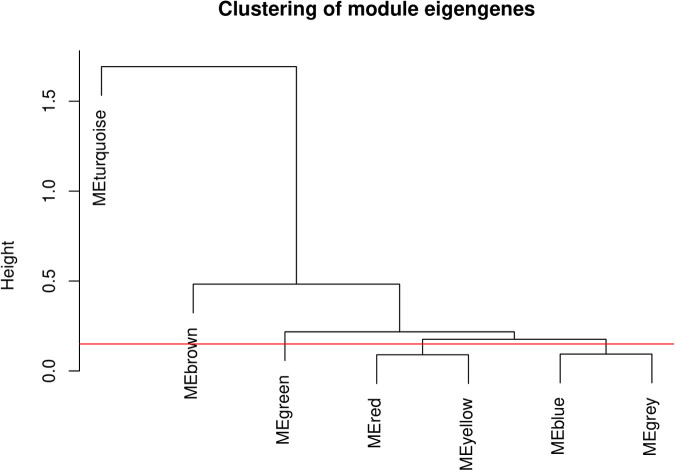
Clustering of module eigengenes. The dendrogram represents the clustering of module eigengenes derived from 10 gene expression studies encompassing 142 samples.

Module eigengenes are representative gene expression profiles for each module and provide a summary of the module’s overall expression pattern. The dendrogram’s branching patterns, as shown in Fig 3, demonstrate the relationships between the modules, where the height reflects the distance or dissimilarity between them.

**Fig 4:**
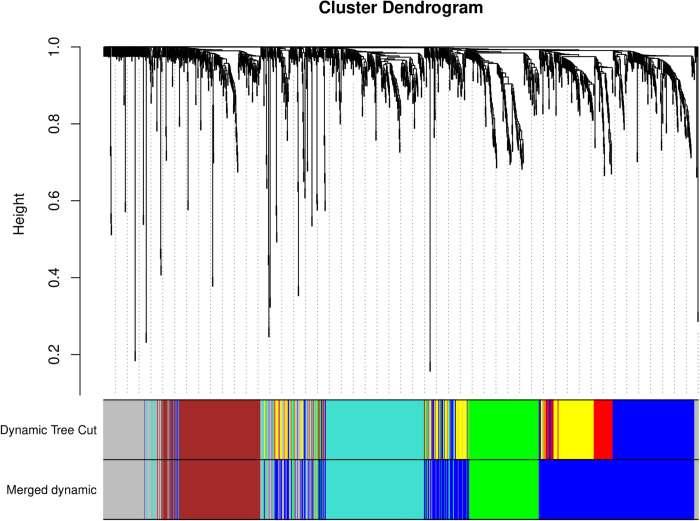
Cluster Dendrogram. The dendrogram branches represent different gene clusters, color-coded to indicate distinct gene modules. The y-axis denotes height, reflecting the level of dissimilarity between clusters. The Dynamic Tree Cut method and Merged dynamic were used to identify and merge modules.

Upon the initial identification of gene modules using the Dynamic Tree Cut algorithm, we observed a set of modules with similar gene expression patterns. To refine our analysis and reduce redundancy, we merged modules that were highly similar, as determined by the correlation of their module eigengenes. The dendrogram generated from the hierarchical clustering of module eigengenes, using average linkage and the dissimilarity measure (dissTOM), provided a visual representation of this similarity. The clear separation of the turquoise module may reflect unique biological functions or regulatory mechanisms that are not shared with the other modules. The closer associations among the brown, green, red, yellow, blue, and grey modules suggest potential overlaps in functional pathways or shared regulatory influences. Modules with a height less than the threshold (0.25) were merged, resulting in a reduced number of modules with distinct and significant expression profiles. The module size ranged from 189 to 536 genes per module, comprising distinct functional gene clusters. Specifically, these modules are contributed as green (n = 189), brown (n = 230), blue (n = 536), turquoise (n = 268), and grey (n = 227) (Fig 4; supplementary file; S2 Table).

**Fig 5:**
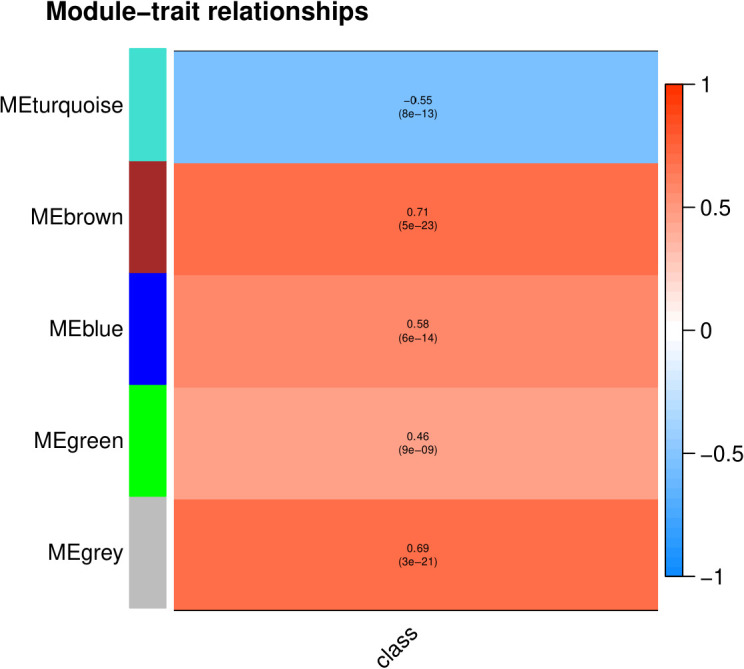
Module-trait relationship. A heatmap illustrating module-trait relationships, where each cell represents the correlation between gene modules (MEturquoise, MEbrown, MEblue, MEgreen, MEgrey) and sample traits. Correlation values range from -1 to 1, indicating negative to positive relationships, respectively. Each cell also displays the correlation coefficient and the significance level (p-value) of the relationship, highlighting the strength and significance of the associations.

The Module-Trait Relationship contains statistical data relating to the correlation between gene expression modules and biotic stress condition (Fig 5). Among these modules, turquoise presented a significant negative connection with abiotic stress (-.055, 8e-13), while four modules showed a notable positive relationship. The module trait relationship was highly positively correlated (0.71, 0.58, 0.46, and 0.69) with low p-values (5e-23, 6e-14, 9e-09, and 3e-21) in the brown, blue, green, and grey modules, respectively. Therefore, these modules were employed for analysis of the membership module and the gene significance.

### Module membership (MM) and gene significance (GS) analysis

**Fig 6:**
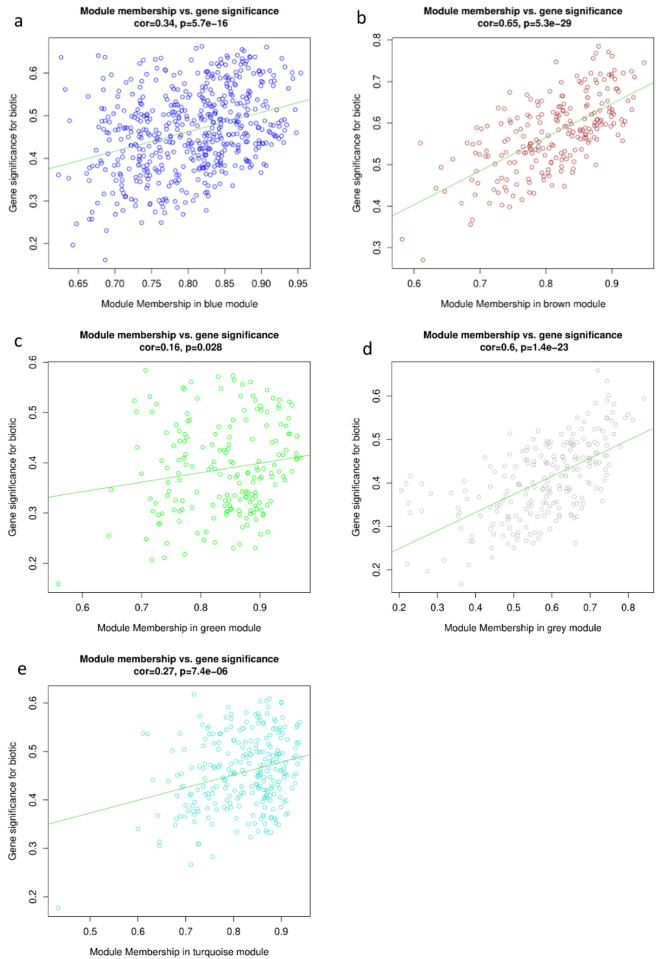
Module membership vs. gene significance. A scatter plot illustrating the relationship between module membership and gene significance for each module. Each point represents a gene, with its position on the x-axis indicating its module membership and on the y-axis indicating its significance for a biotic trait. The correlation coefficient and p-value were provided, indicating a statistically significant correlation between module membership and gene significance.

The membership module and the significance of genes are highly correlated (0.65, and 0.6) with low p-value (5.3e-29, and 1.4e-23) in the brown, and grey modules respectively (Fig 6). The selected 5% of genes in these modules, as shown in Table 1, are hypothesized to play pivotal roles in the stress response due to their high GS and MM.

**Table 1:** Summary of GS and MM values for genes in the Brown and Grey modules. This table includes gene IDs, gene titles, and their respective GS (gene significance) and MM (module membership) values for genes in two different modules.

Module	Gene	Gene Title	Gene Symbol	GS	MM
Brown	Zm.14496.1.A1_at	(S)-beta-macrocarpene synthase		0.7850371	0.8799392
	Zm.14226.1.A1_at	zealexin A1 synthase		0.771352	0.8950371
	Zm.10175.1.A1_at	pathogenesis-related protein 10		0.7682806	0.8756650
	Zm.9486.1.A1_at	CHY1		0.7614173	0.9006719
	ZmAffx.12.1.S1_at	CPP synthase		0.7561538	0.8718411
	Zm.10377.1.A1_at	Peroxidase 64		0.7473302	0.8155571
	Zm.1085.1.A1_a_at	chitinase chem 5		0.7455734	0.9491776
	Zm.16272.1.A1_at	ABC transporter G family member 43		0.7273038	0.9038713
	Zm.2227.1.A1_at	barwin		0.7245416	0.9298464
	Zm.9297.1.A1_at			0.7204611	0.9171292
	Zm.10830.1.S1_at			0.7203482	0.8682409
	Zm.1450.1.S1_at	uncharacterized		0.7185475	0.8828525
Grey	Zm.3129.1.A1_at	polyphenol oxidase I, chloroplastic		0.6593098	0.7202388
	Zm.14845.1.S1_at	uncharacterized		0.6344707	0.7442802
	Zm.2147.1.A1_s_at	uncharacterized		0.594539	0.8416608
	Zm.445.1.S1_at	linoleate 9S-lipoxygenase3 (*lox3*)		0.589756	0.7445617
	Zm.17589.1.A1_at	uncharacterized		0.5843565	0.7135111
	Zm.3303.1.A1_x_at	linoleate 9S-lipoxygenase1 (*lox1*)		0.5813391	0.7623504
	Zm.3303.1.A1_at	linoleate 9S-lipoxygenase1 (*lox1*)		0.5628379	0.7584251
	Zm.17476.1.S1_at	transcription factor MYB8		0.5596002	0.6946274
	Zm.4870.1.A1_x_at	dehydrin		0.5570062	0.7988332
	Zm.4613.1.A1_at	uncharacterized		0.5503393	0.7434835
	Zm.18233.1.S1_at	uncharacterized		0.5500642	0.6671056
	Zm.4870.2.S1_a_at	dehydrin		0.5496261	0.7921241

### Gene ontology and pathway annotation of significant modules

In the top 5% of genes with the highest values in both GS and MM in the brown module, the most significant biological processes are the diterpene phytoalexin biosynthetic process (GO:0051502), the defense response (GO:0006952), the diterpenoid biosynthetic process (GO:0016102), and the defense response to fungus (GO:0050832). The enrichment in these processes suggests that the brown module may be involved in the synthesis of compounds necessary for the defense against pathogens, particularly fungi. For the top 5% of genes with the highest values in both GS and MM in the grey module, the significant GO terms are related to oxylipin biosynthetic process (GO:0031408), lipid oxidation (GO:0034440), and fatty acid biosynthetic process (GO:0006633), implicating this module in lipid metabolism and possibly in the production of signaling molecules related to stress response. The molecular function analysis indicated linoleate 13S-lipoxygenase activity (GO:0016165), which is crucial for the synthesis of oxylipins, a class of compounds known to be involved in plant defense mechanisms. The KEGG pathway analysis highlighted the importance of linoleic acid metabolism (zma00591), which is consistent with the GO results and indicates its involvement in the generation of defense compounds (Table 2).

**Table 2:** This table lists the enriched Gene Ontology (GO) terms and KEGG pathways for genes in the Brown and Grey modules. The categories include Biological Process and Molecular Function, along with corresponding GO terms, gene IDs, p-values, and FDR values.

Module	Category	GO term	Gene	p-value	FDR
**Brown**	Biological Process	GO:0051502~diterpene phytoalexin biosynthetic process	ZM.14226.1.A1_AT, ZMAFFX.12.1.S1_AT	0.001648	0.023076
		GO:0006952~defense response	ZM.14226.1.A1_AT, ZM.10175.1.A1_AT, ZMAFFX.12.1.S1_AT	0.006568	0.045977
		GO:0016102~diterpenoid biosynthetic process	ZM.14496.1.A1_AT, ZMAFFX.12.1.S1_AT	0.013529	0.063136
		GO:0050832~defense response to fungus	ZM.14496.1.A1_AT, ZM.2227.1.A1_AT	0.028914	0.101197
**Grey**	Biological Process	GO:0034440~lipid oxidation	ZM.445.1.S1_AT, ZM.3303.1.A1_AT, ZM.3303.1.A1_X_AT	0.001531	0.021424
		GO:0031408~oxylipin biosynthetic process	ZM.445.1.S1_AT, ZM.3303.1.A1_AT, ZM.3303.1.A1_X_AT	0.003061	0.021424
		GO:0006633~fatty acid biosynthetic process	ZM.445.1.S1_AT, ZM.3303.1.A1_AT, ZM.3303.1.A1_X_AT	0.013852	0.064645
	Molecular Function	GO:0016165~linoleate 13S-lipoxygenase activity	ZM.445.1.S1_AT, ZM.3303.1.A1_AT, ZM.3303.1.A1_X_AT	7.50E-04	0.0045
		GO:0016702~oxidoreductase activity, acting on single donors with incorporation of molecular oxygen, incorporation of two atoms of oxygen	ZM.445.1.S1_AT, ZM.3303.1.A1_AT, ZM.3303.1.A1_X_AT	0.006175	0.018525
	KEGG Pathway	zma00591:Linoleic acid metabolism	ZM.445.1.S1_AT, ZM.3303.1.A1_AT, ZM.3303.1.A1_X_AT	0.005134	0.025672

### Identification of DEGs encoding transcription factors

**Fig 7:**
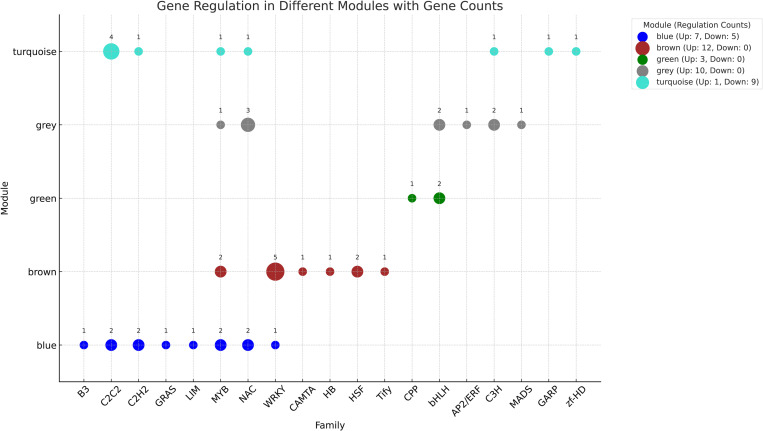
Module TFs. The figure illustrates the regulation of TFs across five distinct modules: blue, brown, green, grey, and turquoise. Each TF family is represented with counts of up-regulated and down-regulated TF members.

According to the results, multiple families of transcription factors (AP2/ERF, B3, bHLH, C2C2, C2H2, C3H, CAMTA, CPP, GARP, GRAS, HB, HSF, LIM, MADS, MYB, NAC, Tify, WRKY, zf-HD) are regulated under biotic stress in maize. The families MYB, NAC, WRKY, and C2C2 demonstrate the highest membership, each consisting of 6 members. Following these, the bHLH family is represented by four members. The blue module showed a total of 12 TFs, with seven genes up-regulated and five genes down-regulated. All 12 TFs in the brown module were up-regulated, indicating a strong activation pattern with no down-regulation observed. Only three TFs were regulated in the green module, all of which were up-regulated. The grey module also showed an up-regulation pattern, with all 10 TFs being up-regulated. Unlike the other modules, the turquoise module had a higher number of down-regulated TFs. Out of the 10 TFs, only one was up-regulated while nine were down-regulated (Fig 7 and Supplementary file; S3 Table).

### Validation of key genes using xgboost

**Fig 8:**
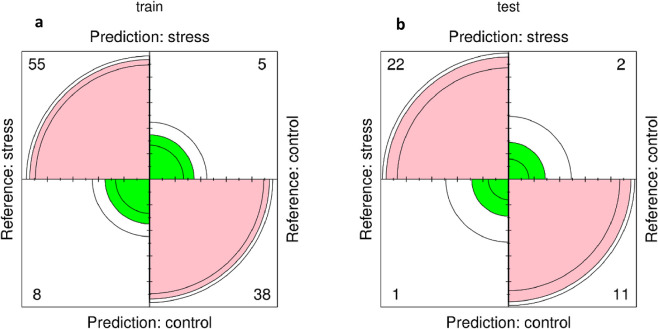
Confusion matrix. This polar plot compares predicted stress and control conditions against reference values. Pink areas indicate stress predictions and green areas indicate control predictions. Numerical values represent sample counts in each category.

Our most intriguing finding is that zealexin A1 synthase, CPP synthase, linoleate 9S-lipoxygenase3 (lox3) and linoleate 9S-lipoxygenase1 (lox1), which were highlighted in the GO ontology analysis, as well as Zm.17476.1.S1_at, encoding the transcription factor MYB8, were in the top 5% of genes with the highest values in both GS and MM in the brown and grey modules. Using xgboost model, the accuracy of 5-gene model was obtained 0.8774 and 0.9167 for training and test data indicating the satisfactory performance of the model. The prediction summary of the model was presented using a confusion matrix (Fig 8).

### Codon usage analysis

The RSCU values for each codon were calculated to identify codon usage bias among the DEGs (Supplementary file; S4 Table). Table 3 highlights the preferred codons for each amino acid based on the highest RSCU values. These preferred codons are used more frequently than others in the differentially expressed genes under biotic stress conditions in maize, indicating a bias that could enhance translational efficiency and gene expression regulation.

**Table 3:** Preferred codons and relative synonymous codon Usage (RSCU) values for amino acids in differentially expressed genes (DEGs) under biotic stress in maize.

amino_acid	codon	RSCU	amino_acid	codon	RSCU
**Arg**	CGC	1.697297	**Gln**	CAG	1.396481
**Gly**	GGC	1.623767	**Glu**	GAG	1.381022
**Ile**	ATC	1.615791	**Thr**	ACC	1.373789
**Val**	GTG	1.520845	**Ala**	GCC	1.345635
**Lys**	AAG	1.476693	**Asn**	AAC	1.289513
**Leu**	CTG	1.462831	**Pro**	CCG	1.216334
**Cys**	TGC	1.456838	**His**	CAC	1.214885
**Phe**	TTC	1.412557	**Asp**	GAC	1.201519
**Tyr**	TAC	1.407763	**Met**	ATG	1
**Ser**	AGC	1.403607	**Trp**	TGG	1

The CAI measures the relative adaptability of the codon usage of a gene to the preferred codon usage of highly expressed genes. The average of CAI values for the DEGs is 0.7793 (Supplementary file; S5 Table). These high CAI values suggest that these DEGs are well adapted for efficient translation in maize.

The ENC value indicates the degree of codon usage bias. The average of ENC values for the DEGs is 48.25 (Supplementary file; S6 Table). These values indicate moderate codon usage bias among the DEGs.

GC content is a measure of the percentage of guanine and cytosine bases in the DNA. The average of GC content values for the DEGs is 0.58 (Supplementary file; S7 Table). These results show a relatively high GC content, suggesting that the DNA of the DEGs may have increased stability and potentially higher melting temperatures.

The average of GC3S values for the DEGs is 0.68 (Supplementary file; S8 Table). High GC3S values suggest a preference for GC-rich codons at the third position, which might be linked to efficient translation and gene expression regulation under biotic stress.

In addition, we examined the codon usage patterns of key genes involved in biotic stress responses, specifically focusing on hub genes such as zealexin A1 synthase, CPP synthase, lox1, lox3, and MYB8, which were identified within the brown and grey modules. Table 4 presents the codon usage metrics, including the CAI, ENC, GC content, and GC3S for these genes. The CAI values of these hub genes range from 0.7746 to 0.9404, with an average value of 0.8697. These CAI values are consistent with the overall DEGs in this study, which also show high CAI values. The ENC values for the hub genes range from 32.64 to 50.66, with an average of 40.45, indicating a moderate level of codon usage bias. This is in line with the DEGs, which also displayed moderate codon usage bias. The GC content for the hub genes ranges from 0.5955 to 0.6814, with an average of 0.6428, which is slightly higher than the average GC content of the DEGs (0.58), suggesting a preference for GC-rich codons. The GC3S values for the hub genes range from 0.7203 to 0.9821, with an average of 0.8731, indicating a strong preference for GC-rich codons at the third codon position.

**Table 4:** Codon usage metrics for five Key genes identified in biotic stress response modules.

Microarray probes	Gene ID	CAI	ENC	GC	GC3S
**ZM.14226.1.A1_AT**	Zm00001eb222660	0.843717	44.0851	0.614625	0.827083
**ZMAFFX.12.1.** S1 **_AT**	Zm00001eb021200	0.774593	50.66316	0.595524	0.720253
**ZM.445.1.** S1 **_AT**	Zm00001eb054040	0.940398	32.64803	0.67001	0.982116
**ZM.3303.1.A1_AT**	Zm00001eb144960	0.927533	33.98904	0.652827	0.954377
**Zm.17476.1.** S1 **_at**	Zm00001eb138920	0.862608	40.87493	0.681351	0.881818

**Fig 9:**
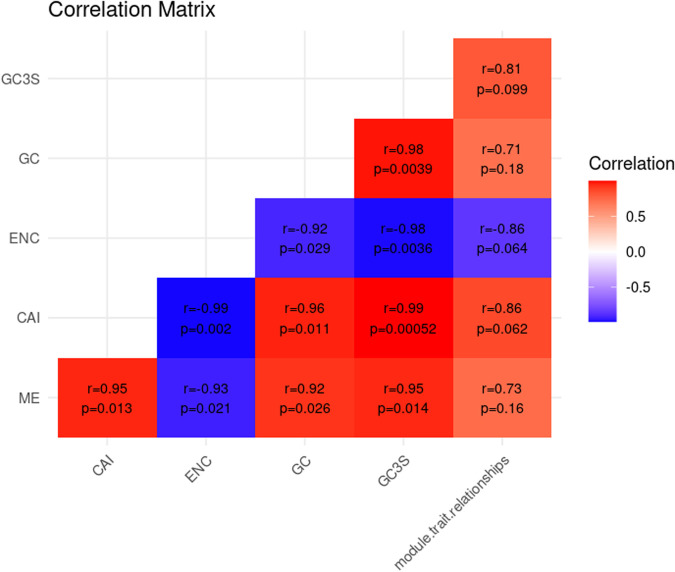
Correlation heatmap. Correlation matrix illustrating the relationships between codon usage indices (CAI, ENC, GC, and GC3S), MEs, and module-trait relationships. The values indicate the strength of the correlation (r) along with the corresponding p-values. Positive correlations are shown in red, while negative correlations are in blue.

Moreover, the correlation analysis revealed significant relationships between MEs, trait-module relationships, and codon usage indices. Notably, strong positive correlations were observed between the CAI, GC content, and GC3S with the trait-module relationships. Furthermore, a negative correlation was found between the ENC and the trait-module relationships (Fig 9).

## Discussion

By performing a meta-analysis of gene expression responses in maize to biotic stress, we aimed to uncover previously unidentified genes and pathways involved in the maize defense mechanisms across 10 studies with 142 samples. After rigorous pre-processing and correcting for batch effects, we identified 1449 DEGs that show significant changes (adjusted p-value < 0.05) between control and biotic stress conditions.

WGCNA was then utilized to analyze the co-expression patterns of genes implicated in maize defense against biotic stresses. The selection of the appropriate soft-thresholding power is a balance between achieving scale-free topology (as close to 1 as possible) and maintaining a high mean connectivity. According to the scale independence plot, a soft-thresholding power of 14 was considered an appropriate choice for this analysis as it met the scale-free topology criterion.

The gene modules identified through dendrogram analysis provided valuable insights into the biological pathways and networks involved. The co-expression patterns suggest that genes within the same module may be co-regulated or participate in related biological processes. This finding is pivotal for understanding the genetic basis of biotic stress response. Further investigation into these modules unveiled key regulatory genes and potential targets for enhancing biotic stress tolerance.

The modular organization of gene expression patterns underscores the complexity of gene regulation and emphasizes systems biology approaches to elucidate these intricacies. Merging closely related modules is a critical step toward simplifying the complexity of gene expression data and enhancing results interpretability. This merging process consolidates genes that are not only co-expressed but likely co-regulated or part of the same biological pathways. This process reduces the noise in the data, potentially uncovering the more robust and biologically relevant signals. The merged modules were further analyzed for their association with biotic stress response and for the identification of key driver genes.

By focusing on these refined modules, we can direct our subsequent analyses and experiments to those genes and pathways that are most likely to yield meaningful insights into the biological processes under study. Further functional enrichment analysis of these modules elucidated specific biological processes and pathways involved, aiding in the identification of novel targets for the development of biotic stress tolerance or biomarkers of biotic stress response.

We assessed the significance of the modules by calculating their correlation strengths with the experimental conditions (stress vs. control). As shown in the heatmap (Fig 5), the modules showed different correlations with the conditions. The modules were ranked based on the absolute value of their correlation strengths. This ranking reflects the relevance of each module in response to the experimental conditions. The MEbrown module showed the highest correlation with the stress condition (correlation = 0.71, p-value = 5e-23). The MEgrey module was the second most significant, with a correlation of 0.69 (p-value = 3e-21). This was followed by the MEblue, MEturquoise, and MEgreen modules with correlations of 0.58, -0.55, and 0.46, respectively.

According to the module-trait relationship, a strong correlation was found in the brown and grey modules with the biotic stress condition. These modules likely consist of genes functioning synergistically in stress response pathways. The selected 5% of genes in brown and grey modules could be central in regulatory or pathway-specific roles, potentially serving as key targets for genetic manipulation or further functional studies.

According to the GO results, the identification of these processes and pathways suggests a complex response to biotic stress in maize, involving multiple layers of defense. The enrichment of the brown module in phytoalexin biosynthesis indicates activation of chemical defenses commonly used by plants to prevent pathogen progression. The presence of the ZM.14226.1.A1_AT and ZMAFFX.12.1.S1_AT genes in several significant GO terms suggests that these genes may play a key role in the stress response.

Similarly, the genes in the grey module are associated with the synthesis of oxylipins and related compounds, which can serve as signals to activate defense responses or directly deter pathogens. Oxylipins are oxidized metabolites of polyunsaturated fatty acids (PUFAs), such as linoleic acid (LA) and α-linolenic acid (ALA) [24].

The overlap of genes such as ZM.445.1.S1_AT and ZM.3303.1.A1_AT in several significant GO terms within the grey module also highlights the multifunctional nature of these genes in the stress response. The high fold enrichment values for these genes in lipid-related processes highlight their potential importance in the defense mechanism.

Our most intriguing finding is that the genes ZM.14226.1.A1_AT (zealexin A1 synthase), ZMAFFX.12.1.S1_AT (CPP synthase), ZM.445.1.S1_AT (linoleate 9S-lipoxygenase3 (lox3)), and ZM.3303.1.A1_AT (linoleate 9S-lipoxygenase1 (lox1)), highlighted in the GO ontology analysis, were among the top 5% of genes with the highest values in both GS and MM in the brown and grey modules. This research positions these four genes as prime candidates for genetic manipulation in *Zea mays* to improve tolerance to biotic stresses. Also, in our previous finding [25], zealexin A1 synthase was among the top genes differentially upregulated against biotic stress in maize.

Zealexin A1 synthase is responsible for the production of zealexin A1, which is part of a group of novel acidic sesquiterpenoids that constitute a dominant class of phytoalexins in maize, highlighting its significance in plant immunity and response to biotic stress. The production of zealexin A1 is induced in response to insect attack, with total zealexin levels significantly higher than those caused by mechanical damage alone within 4 days of stem herbivory [26].

CPP synthases, including class II diterpene synthases such as ZmCPS3 and ZmCPS4 in maize, are crucial enzymes involved in producing specialized diterpenoid metabolites that help the plant defend against both biotic and abiotic stresses [27]. Diterpenoids constitute a significant class of phytoalexins in plants. Their biosynthesis is tightly regulated by the coordinated action of CPP synthases and KSL enzymes, leading to the production of various diterpenoid scaffolds that contribute to plant defense against biotic and abiotic stresses (Jeandet et al., 2014; Valletta et al., 2023; Kariya et al., 2023).

9-LOXs such as the pepper 9-lipoxygenase gene CaLOX1 [28] and the tea plant 9/13-lipoxygenase CsLOX1 [29] positively regulate defense and cell death responses to microbial pathogens and insect pests in plants. The 9-LOX pathway produces oxylipins, which serve as signaling molecules to activate various defense mechanisms against biotic stresses [30–32].

The production of phytoalexins is often induced by the presence of oxylipins, which can act as signaling molecules to activate the biosynthesis of these antimicrobial compounds. For example, oxylipin 12-oxo-phytodienoic acid (OPDA) has been shown to induce the production of phytoalexins in plants [33,34]. Other oxylipins such as jasmonates can also trigger phytoalexin biosynthesis as part of the plant’s defense response [35]. In other words, oxylipins can serve as precursors or initiators that stimulate the production of antimicrobial phytoalexins in plants as an important defense mechanism against pathogens and other stresses.

These results provide evidence for a synergistic defense response in maize resulting from the combined effect of oxylipin signaling and phytoalexin production. In particular, oxylipins initiate defense responses, while phytoalexins directly combat microbial threats, which together enhance maize tolerance to biotic stresses. Consequently, this multi-layered defense mechanism strengthens the overall tolerance of maize. Oxylipins function as signaling molecules that trigger the biosynthesis of phytoalexins, which in turn act as potent antimicrobial compounds to protect plants. Accordingly, this collaboration between these two plant metabolites constitutes a vital component of the overall defense system in maize.

The blue and brown modules, with a high number of up-regulated TFs, may be indicative of active biological processes that require increased gene expression. In contrast, the turquoise module, which has a majority of down-regulated TFs, might be involved in processes where suppression of gene activity is critical. Zm.17476.1.S1_at encoding transcription factor MYB8 was among the top 5% of genes with the highest values in both GS and MM in the grey module. Recently, several authors have indicated the essential role of MYB TFs in primary and secondary metabolism, hormone synthesis, signal transduction, and against pathogen infection [36–38]. The host defense against *Ralstonia solanacearum* in pepper was positively regulated by MYB TF [39]. The MYB TF in rice plays a significant role in broad-spectrum blast resistance [40]. Also, MdMYB30 is involved in regulation of cuticular wax accumulation resulting in resistance enhancement against fungal pathogen *Botryosphaeria dothidea* in apple [41]. Simultaneously, MdMYB73 plays a role in regulation of the salicylic acid pathway confering resistance in apple against the *Botryosphaeria dothidea* [42]. MYB TF has been found to activate the expression level of *stilbene synthase* gene in the chinese wild grapevine (*Vitis davidii*) and positively regulates the defense response against invading pathogens [38]. However, the role of MYB TFs in host defense response against biotic stress in maize is unknown. Considering that the transcription factor MYB8, encoded by the gene Zm.17476.1.S1_at, ranked among the top 5% of genes with the highest values in both Gene Significance and Module Membership within the grey module, it is plausible to propose that MYB8 might play a significant role in maize’s response to biotic stress.

Bias in codon usage also plays a role in the plant’s ability to respond to stress. Studies have shown that stress-responsive genes in plants, such as rice, have specific codon usage patterns. For instance, genes that are upregulated under stress tend to have higher GC content and prefer codons with C or G at the third nucleotide position. This bias is believed to be a result of natural selection, optimizing these genes for efficient expression under stress conditions [43,44]. In addition, codon optimization can be used to increase the expression of transgenes in plants, making them more resistance to stress. By designing genes with codons that match the tRNA pool of the host plant, researchers can improve the expression and functionality of these genes, aiding in the development of stress-tolerant plant varieties [43,45].

The RSCU showed a clear bias in codon usage among the DEGs. Codons with RSCU values greater than 1.0 suggest a preference that can influence translational efficiency. For instance, the preferred codons for amino acids such as CGC for arginine and GGC for glycine, with RSCU values of 1.697 and 1.623 respectively, indicate a strong bias. These biases could enhance the efficiency of translation and regulation of gene expression under stress conditions.

The CAI is a measure of how well the codon usage of a gene matches the preferred codon usage of highly expressed genes. The average CAI value of 0.7793 for the DEGs suggests that these genes are well adapted for efficient translation in maize. High CAI values are indicative of potential higher expression levels, which is crucial for the rapid and effective response of maize to biotic stress.

The average ENC value of 48.25 among the DEGs indicates a moderate codon usage bias. This moderate bias suggests a balanced use of codons, which may be optimal for maintaining both efficiency and flexibility in gene expression under varying environmental conditions.

The GC content, representing the percentage of guanine and cytosine bases in the DNA, has significant implications for the stability of the DNA molecule. The average GC content of 0.58 among the DEGs indicates a relatively high GC content, suggesting increased stability and potentially higher melting temperatures of the DNA. This higher stability can be advantageous under stress conditions, ensuring that the genetic material remains intact and functional.

The average GC3S value of 0.68 among the DEGs suggests a strong preference for GC-rich codons at the third position. This preference may be linked to efficient translation and robust gene expression regulation under biotic stress, contributing to the resilience of maize.

The codon usage analysis added another layer of understanding, suggesting that codon bias may influence gene expression and translational efficiency during biotic stress response in maize. The high CAI and GC content values for many DEGs suggest that these genes are optimized for efficient translation and stability.

In this study, all DEGs identified through meta-analysis were utilized to construct a co-expression network and to conduct codon usage analysis. These two analyses are designed to provide complementary insights: WGCNA identifies biologically meaningful modules and key pathways linked to biotic stress responses, while codon usage analysis explores whether DEGs demonstrate adaptive codon preferences that enhance translational efficiency and regulatory dynamics under stress conditions. Our analysis of codon usage patterns among all DEGs, including those from the identified modules, showed a marked preference for codons with high CAI, GC, and GC3S content. Thus, while WGCNA reveals the coordinated expression of genes within biological networks, codon usage analysis enhances our understanding by uncovering evolutionary adaptations in translational control mechanisms throughout all DEGs. This integrated approach provides a comprehensive perspective on the transcriptional and translational landscape in response to biotic stress. It establishes a solid framework for future genetic improvement strategies, focusing on both transcriptional and translational optimization of key genes. Hub genes such as zealexin A1 synthase, CPP synthase, lox1, lox3, and MYB8 are expected to play pivotal roles in stress responses. To explore this further, we conducted a detailed analysis of the codon usage patterns specific to the hub genes identified within the brown and grey modules. The hub genes displayed codon usage patterns that align with those of the overall DEG population, characterized by high CAI values and a preference for GC-rich and GC3S codons (as illustrated in Table 4). These findings suggest that translational optimization is a common characteristic shared by both the hub genes and the general DEG set, emphasizing the significance of codon usage bias in facilitating effective responses to stress.

To explore the connection between the significance of modules and codon usage patterns, we analyzed the correlation of codon usage metrics for genes within the significant modules with MEs of corresponding modules and trait-module relationships. As illustrated in Fig 9, we identified significant correlations between MEs and codon usage indices, such as the CAI, ENC, GC content, and GC3S. These results highlight a strong and meaningful link, underscoring how codon usage affects gene expression and its association with specific modules. Additionally, we found strong positive correlations between CAI, GC content, and GC3S with the trait-module relationships. This suggests that as these indices increase, the strength of the association between modules and traits also strengthens, indicating that codon usage positively influences gene expression within these modules. In contrast, there was a strong negative correlation between ENC and the trait-module relationships, suggesting that higher ENC values correspond to weaker associations between modules and traits. While there are notable correlations between codon usage metrics and trait-module relationships, the lack of statistical significance suggests a need for further investigations and more detailed analyses to understand these dynamics better.

## Conclusions

The application of WGCNA provided valuable insights into the complex gene networks underlying the biotic stress response in maize. Moreover, the identification of key co-expression modules and hub genes, such as zealexin A1 synthase, CPP synthase, lox3, lox1, and MYB8 provides promising targets for future maize improvement strategies. Using this knowledge, researchers and breeders can develop more effective approaches to improve maize tolerance to biotic threats, ultimately contributing to global food security. However, while this study has provided valuable insights, the identified hub genes, such as zealexin A1 synthase, CPP synthase, lox3, lox1, and MYB8 should be further validated through targeted experiments, such as gene expression analysis, gene knockout studies, and functional characterization. This validation will help confirm their roles in the biotic stress response and their potential for genetic manipulation. Additionally, the analysis was based on the available microarray gene expression data, which may not capture the full complexity of the transcriptional changes that occur during biotic stress. Therefore, combining gene expression data with other omics datasets could provide a more comprehensive understanding of the regulatory mechanisms and signaling pathways involved in the biotic stress response of maize. By addressing these limitations, future studies can build on the findings of this work to further elucidate the intricate gene networks underlying the biotic stress response in maize and pave the way for more targeted and efficient maize improvement strategies. Moreover, this study provided valuable insights into the codon usage patterns. Understanding codon usage patterns and related metrics such as RSCU, CAI, ENC, GC content, and GC3S can guide the design of synthetic genes that are optimized for expression in maize, enhancing their performance in biotechnological applications. By identifying and manipulating genes with favorable codon usage, it is possible to develop maize varieties with improved stress tolerance and productivity. Optimizing codon usage can lead to more efficient protein production, which is critical for the rapid response to environmental stresses and for improving overall plant health and yield.

## Supporting information

S1 Table Comprehensive analysis of maize response to biotic stress identified 1449 differentially expressed genes across 10 studies comprising 142 samples. (XLSX)

S2 Table The WGCNA identified 5 distinct gene expression modules, with module sizes ranging from 189 to 536 genes. (XLSX)

S3 Table DEGs encoding transcription factors and their corresponding Ensembl ID, Family, Rgulation, and Module. (XLSX)

S4 Table RSCU values for amino acids in differentially expressed genes (DEGs) under biotic stress in maize. (XLSX)

S5 Table CAI values for the differentially expressed genes (DEGs) under biotic stress in maize. (XLSX)

S6 Table ENC values for the differentially expressed genes (DEGs) under biotic stress in maize. (XLSX)

S7 Table GC values for the differentially expressed genes (DEGs) under biotic stress in maize. (XLSX)

S8 Table GC3S values for the differentially expressed genes (DEGs) under biotic stress in maize. (XLSX)
